# Inhibitory effects of *Cinnamomum burmannii* Blume stem bark extract and *trans*-cinnamaldehyde on nasopharyngeal carcinoma cells; synergism with cisplatin

**DOI:** 10.3892/etm.2013.1041

**Published:** 2013-04-02

**Authors:** MAELINDA DAKER, VOON YEE LIN, GABRIEL AKYIREM AKOWUAH, MUN FEI YAM, MARIAM AHMAD

**Affiliations:** 1 Molecular Pathology Unit, Institute for Medical Research, Jalan Pahang, Kuala Lumpur 50588;; 2 Faculty of Pharmaceutical Sciences, UCSI University, Kuala Lumpur 56000;; 3 Faculty of Pharmaceutical Sciences, Universiti Sains Malaysia, Minden, Penang 11800, Malaysia

**Keywords:** *Cinnamomum burmannii*, cell proliferation, cisplatin, nasopharyngeal carcinoma, synergism, *trans*-cinnamaldehyde

## Abstract

Nasopharyngeal carcinoma (NPC) is a malignancy that occurs in the epithelium of the nasopharynx. The standard treatment of NPC patients with locoregionally advanced stages is problematic and is often associated with toxicities. Therefore, it is essential to screen for naturally occurring compounds with strong apoptosis-inducing activity and minimal toxicity. This study investigated the effects of the standardized methanol extract of *Cinnamomum burmannii* Blume stem bark and its main constituent, *trans*-cinnamaldehyde (TCA), on human NPC cell lines. The content of TCA in *C. burmannii* methanol extract was standardized to be 13.61% w/w by means of gas chromatography-mass spectrometry (GC-MS). NPC cell proliferation was clearly inhibited within 24 h of treatment, with TCA exhibiting greater activity than the methanol extract. TCA was more active against NPC cells compared with cisplatin. There was a pronounced downregulation of the proliferation markers, Ki67 and proliferating cell nuclear antigen (PCNA) in the TCA-treated cells; while morphological observation indicated the induction of apoptosis. Caspase activation and prominent DNA damage, which are markers of apoptosis induction were detected. TCA demonstrated the ability to scavenge nitric oxide. The simultaneous combination of TCA and cisplatin produced synergistic anti-proliferative effects. Collectively, these data indicate the potential use of TCA for the treatment of NPC.

## Introduction

Nasopharyngeal carcinoma (NPC) is a squamous cell carcinoma that develops in the epithelial lining of the nasopharynx. The disease occurs among individuals in Southern China and North Africa, as well as Inuits from Alaska and Bidayuhs from East Malaysia (1,2). Patients with locoregionally advanced stages are treated by concurrent chemotherapy and radiotherapy with or without adjuvant chemotherapy. Unfortunately, conventional cancer therapies are often associated with toxicities. Alternative approaches that selectively kill tumor cells whilst having weaker or negligible effects on healthy tissues are required (3).

*Cinnamomum burmannii* Blume (*Lauraceae*) is a tree-like shrub that is native to Southeast Asia and Indonesia (4). The aromatic bark is used for medicine and making spices for the flavor industry (5). *Trans*-cinnamaldehyde (TCA) has been identified as one of the bioactive compounds in *C. burmannii*(6). Numerous reports on the anti-cancer activities of *Cinnamomum* have been published (7–9). Additionally, studies have demonstrated that TCA inhibits cell proliferation and induces cell apoptosis (10–13). Despite the extensive research on the effect of TCA on a variety of cell lines, studies concerning its anti-proliferative effect on NPC cells are limited. Therefore, the present study addresses the anti-neoplastic potential of the methanol extract of the *C. burmannii* stem and its constituent, TCA, on HK1 and C666-1 NPC cell lines. We also explored the potential of combining TCA with conventional cytotoxic chemotherapy for more successful elimination of NPC cells.

## Materials and methods

### Chemicals and reagents

Curcumin and TCA were purchased from Sigma-Aldrich (St. Louis, MO, USA). All other solvents, reagents and chemicals used were of analytical or high-performance liquid chromatography (HPLC) grade from Sigma-Aldrich or Merck (Darmstadt, Germany).

### Plant material and extraction

The stem bark of *C. burmannii* was collected from Agroforestry Garden, Sumatra, Indonesia and was authenticated by Mr Onrizal S. Hut, Forestry Department, Universitas Sumatera Utara, Indonesia. A voucher specimen (BO.0032998) was stored for future reference. The stem bark was air-dried and ground into a powder. The powdered plant material (250 g) was extracted with methanol (2.5 liters) using Soxhlet apparatus. The extract was concentrated to dryness under reduced pressure and temperature (40°C). The yield (w/w) of the extract from the dry stem bark was 9.8%. The extract was stored in a desiccator at room temperature until analysis.

### Gas chromatography-mass spectrometry (GC-MS) of the extract

GC-MS was performed using the Agilent 7000 GC-MS/MS Triple Quadrupole mass spectrometer (Agilent Technologies, Inc., Santa Clara, CA, USA) operated under the full scan mode. Stock solution (1 mg/ml) of the authentic TCA standard was prepared in methanol. Calibration standards were prepared from the stock solution at a concentration range of 1 to 100 *μ*g/ml. One milligram portions of methanol extract were weighed and dissolved in 1 ml methanol. The solution was diluted accordingly to fit into the calibration range prior to analysis. The GC was operated at an initial temperature of 73°C, then increased by 50°C/min until a final temperature of 280°C was reached. It was held at this temperature for 1 min. The column used was a DB-5 column (length, 30.0 m; diameter, 250 *μ*m) with helium as the carrier gas. The injection volume was 2 *μ*l, operated in split mode with a ratio of 1:10.

### Cell culture

The NPC cell line, HK1 (14), was maintained in the exponential growth phase in RPMI-1640 medium (Gibco Life Technologies, Carlsbad, CA, USA) supplemented with 10% heat-inactivated fetal calf serum (FCS; Gibco Life Technologies), 10 U/ml penicillin (Gibco Life Technologies) and 10 *μ*g/ml streptomycin (Gibco Life Technologies) at 37°C in a 5% CO_2_ humidified atmosphere. C666-1 (15), also an NPC cell line, was maintained similarly; however, the FCS concentration was increased to 15%. The immortalized human skin keratinocyte, HaCaT, represented normal cells. HaCaT (16) was purchased from CLS Cell Lines Service (Eppelheim, Germany) and was maintained in Dulbecco’s modified Eagle’s medium (DMEM; Gibco Life Technologies) supplemented with 10% FCS. Tests for detection of mycoplasma using an e-Myco™ Mycoplasma polymerase chain reaction (PCR) Detection kit (Intron Biotechnology, Seoul, Korea) were conducted routinely and contamination-free cells were used throughout this study.

### Cell viability assay

The effect of the methanol extract and TCA was determined using the CellTiter 96^®^ AQueous One Solution Cell Proliferation (MTS) assay (Promega Corporation, Madison, WI, USA). Cells (5,000–15,000 cells/well) were seeded into 96-well microtiter plates in 100 *μ*l culture medium and then incubated. Old medium from the exponentially growing cells was replaced with medium containing the desired concentrations of test substances for 24 h at 37°C in a 5% CO_2_ atmosphere. The concentrations used for the study were 100–450 *μ*g/ml methanol extract or 2–16 *μ*g/ml TCA. The methanol extract was dissolved in dimethyl sulfoxide (DMSO; Sigma-Aldrich) with a final concentration of 0.5%. Vehicle control cultures received DMSO alone. Cells treated with cisplatin served as the positive control. Absorbance at 490 nm was read using the EnVision multilabel plate reader (Perkin-Elmer, Waltham, MA, USA) and the non-specific absorbance measured at 630 nm was subtracted. Wells containing the appropriate culture medium without cells served as the blank measurement. Cell viability was estimated as the percentage of population growth compared with control cells (arbitrarily assigned as 100% viable). The IC_50_ values, defined as the concentration that inhibited the cell growth by 50% relative to that of control cells, was graphically obtained from the dose-response curves. All experiments were performed in triplicate. The results were expressed as the mean ± standard deviation (SD).

### xCELLigence cell proliferation assay

Cells (5,000–15,000 cells/well) were seeded into E-Plate 16 devices (ACEA Biosciences Inc., San Diego, CA, USA) containing 100 *μ*l culture medium. Upon reaching the logarithmic phase, the culture medium was aspirated and replaced with culture medium containing either 100 *μ*g/ml methanol extract or 4–8 *μ*g/ml TCA. The methanol extract was dissolved in DMSO, to a final concentration not exceeding 0.5%. Vehicle control cultures received DMSO alone. Cells treated with cisplatin served as the positive control. Dynamic monitoring of the growth inhibition pattern was determined by the impedance-based xCELLigence system (Roche Applied Science, Mannheim, Germany) for 24 h at 37°C in a humidified atmosphere of 5% CO_2_. The cell index, automatically calculated from the change in electrical impedance as the living cells interacted with electrodes in the E-Plate wells, correlated with the number of cells, viability and/or cytotoxicity over time.

### Quantitative real time PCR

Cells were grown to near confluence, then were either treated with 4–8 *μ*g/ml TCA or left untreated for 24 h. Total RNA was extracted with TRIzol reagent (Invitrogen Life Technologies, Carlsbad, CA, USA). DNase I (Promega Corporation) treatment was performed according to the manufacturer’s instructions. cDNA was synthesized from 2 *μ*g total RNA using the High Capacity cDNA Reverse Transcription kit (Applied Biosystems, Foster City, CA, USA), then amplified using Power SYBR-Green Master Mix (Applied Biosystems) in the Applied Biosystems 7500 Fast Real Time PCR system and analyzed with 7500 software v.2.0.4. The primers for Ki67 were as follows: Ki67 forward: 5′-ATGGAAGCGTCACGGGAA-3′ and reverse: 5′-TCTTCCGCAGGTTCAATTCTT-3′. β-actin (ACTB) and peptidylprolyl isomerase A (PPIA) were amplified as the internal controls using the following primers: ACTB forward: 5′-TCATCACCATTGGCAATGAG-3′ and reverse: 5′-CACTGTGTTGGCGTACAGGT-3′; PPIA forward: 5′-GGCCAGGCTCGTGCCGTTTT-3′ and reverse: 5′-TGCTGTCTTTGGGACCTTGTCTGC-3′.

### Western blotting

Cells were grown to near confluence, then the old media was aspirated and replaced with media with or without 4–8 *μ*g/ml TCA. The cells were then re-incubated. Cells were lysed in 1X RIPA lysis buffer and then boiled for 10 min. The quantity of protein in the cell lysate was determined by protein assay (Bio-Rad, Hercules, CA, USA). The protein (10 *μ*g) was resolved in NuPAGE^®^ Novex Bis-Tris Mini Gels (Invitrogen Life Technologies) and electro-transferred to polyvinylidene fluoride (PVDF) membranes (Millipore, Bedford, MA, USA). The membranes were blocked with 5% skimmed milk and incubated overnight with primary antibodies diluted in 5% skimmed milk. Primary antibodies (Cell Signalling Technology, Inc., Danvers, MA, USA) used were monoclonal mouse anti-glyceraldehyde 3-phosphate dehydrogenase (GAPDH), monoclonal mouse anti-proliferating cell nuclear antigen (PCNA) and rabbit anti-phospho-histone H2AX (Ser139). The secondary antibody reaction was performed with anti-mouse or anti-rabbit horse-radish peroxidase-conjugated IgG (Promega Corporation). Western Lightning^®^ Plus enhanced chemiluminescence (ECL) substrate (Perkin-Elmer) and autoradiography were used for visualization of protein expression. Densitometric analysis of X-ray films was performed on an Alpha Imager System (ProteinSimple, Santa Clara, CA, USA) using Alpha View software. The band intensities were normalized with respect to the GAPDH product.

### Morphological observation

Cells were seeded at a density of 5,000–15,000 cells/well into 96-well microtiter plates and were left to grow exponentially at 37°C in a 5% CO_2_ atmosphere. Then, the cells were treated with 4–8 *μ*g/ml TCA or 20–30 *μ*g/ml cisplatin. After 24 h incubation, morphological changes were observed under a Leica DM IRB (Leica Microsystems, Wetzlar, Germany) inverted microscope.

### Caspase-3/7 activity assay

Cells were seeded and treated as described above. After the appropriate exposure times, caspase-3/7 activation was measured using the ApoTox-Glo™ Triplex assay (Promega Corporation) kit according to the manufacturer’s instructions. The luminescence signal was read using the EnVision multilabel plate reader (Perkin-Elmer).

### Apoptosis analysis assay

HK1 cells were seeded in 10-cm culture dishes to reach confluence overnight. Following this, the cells were treated with 8 *μ*g/ml TCA, 30 *μ*g/ml cisplatin or left untreated (as the control). All culture dishes were re-incubated for a further 24 h. Apoptosis was determined with a FACSCalibur flow cytometer (BD Biosciences, San Jose, CA, USA), using the fluorescein isothiocyanate (FITC) Annexin V apoptosis detection kit (BD Pharmingen, San Diego, CA, USA), according to the manufacturer’s instructions.

### Combined drug analysis

For combined drug analysis, a non-fixed ratio combination of TCA and cisplatin was evaluated. Drug dilutions and combinations were made in RPMI-1640 medium immediately before use. Following drug addition, the 96-well microtiter plates were incubated for 24 h and the MTS assay was performed to determine cell viability. Drug interaction was determined by the combination-index (CI) and isobologram methods (17). Dose-response curves, dose-effect analysis and CI for the combination treatment group were generated using CalcuSyn 2 software (Biosoft, Cambridge, UK). CI >1 implies antagonism, CI =1 is additive and CI <1 implies synergism.

### Nitric oxide radical inhibition assay

The scavenging effect of the extracts on nitric oxide radicals was measured according to the method of Rao (18). Curcumin was used as a positive reference compound. Measurements were performed six times. The percentage inhibition of nitric oxide radical generation was calculated. The inhibitory concentration at which 50% of the nitric oxide radical was scavenged by test samples (IC_50_) was determined from the graph of inhibition percentage against sample concentration.

### Statistical analysis

Statistical analysis was performed using SPSS 12.0 statistical software for Windows (SPSS Inc., Chicago, IL, USA). Differences between mean values were evaluated with the Student’s t-test or one-way analysis of variance (ANOVA) and Tukey’s or Dunnett’s T3 post hoc analysis. P<0.05 was considered to indicate a statistically significant difference.

## Results

### GC-MS analysis

**Figure 1:**
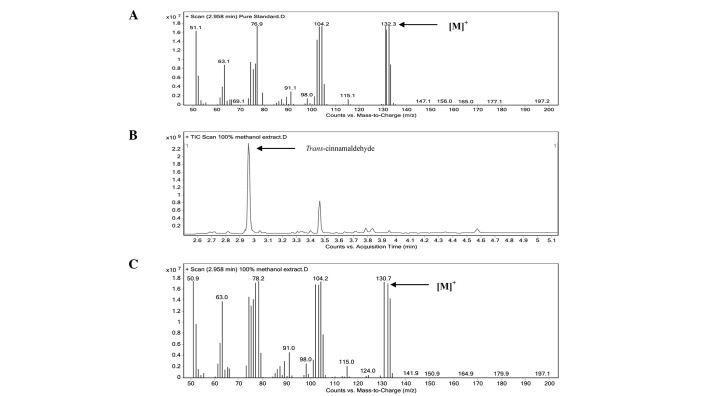
Results of gas chromatography-mass spectrometry (GC-MS). (A) Mass spectrum of authentic *trans*-cinnamaldehyde standard; (B) total ion chromatogram of the methanol extract of *Cinnamomum burmannii* Blume stem bark and (C) mass spectrum for the peak corresponding to *trans*-cinnamaldehyde from the methanol extract. [M]^+^, molecular ion.

The marker (TCA) was identified in the extracts by comparing the retention time and mass spectrum with that of the authentic standard. The mass spectrum of the authentic TCA standard is shown in Fig. 1A. The total ion chromatogram (TIC) of the methanol extract and mass spectrum of the peak-matching TCA are shown in Fig. 1B and C, respectively. TCA was the major peak in the extract. The TCA component of the extracts presented the [M]^+^ ion at 132 m/z, which corresponded to 132.3 m/z observed in the mass spectrum of the authentic TCA standard, confirming a similar identity. The content of TCA in the methanol extract was standardized to 13.61% w/w by an external standardization method.

### Determination of viable cells

**Figure 2:**
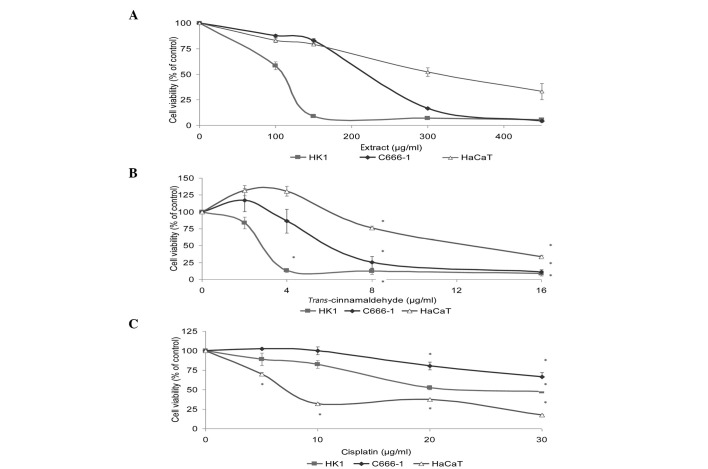
Concentration-dependent viability of HK1, C666-1 and HaCaT cells in the presence of (A) methanol extract of *Cinnamomum burmannii* Blume stem bark, (B) *trans*-cinnamaldehyde and (C) cisplatin. Values expressed as mean ± standard deviation (SD) are the mean of three independent experiments. In (A), the percent viability at all concentration points was significantly different from the untreated control (P<0.05). ^*^P<0.05, compared with the untreated control group.

We assessed the effects of the standardized methanol extract and TCA on the HK1 and C666-1 NPC cell lines. NPC cell growth was inhibited (Fig. 2A and B) in a concentration-dependent manner. TCA at 16 *μ*g/ml caused almost 90% inhibition of cell growth (Fig. 2B). To determine whether normal cell growth was also inhibited, we investigated cell viability in the immortalized human skin keratinocyte, HaCaT. The order of cells affected by the activity of test substances was HK1 > C666-1 > HaCaT. Cisplatin (positive control drug) demonstrated a much higher toxic effect in normal cells, affecting HaCaT to a greater extent than the NPC cell lines (Fig. 2C). TCA was quite potent against NPC cells with IC_50_ values lower than that of cisplatin, demonstrating far higher efficacy than cisplatin against the NPC cells (Table I).

### Dynamic monitoring of cell proliferation

**Figure 3:**
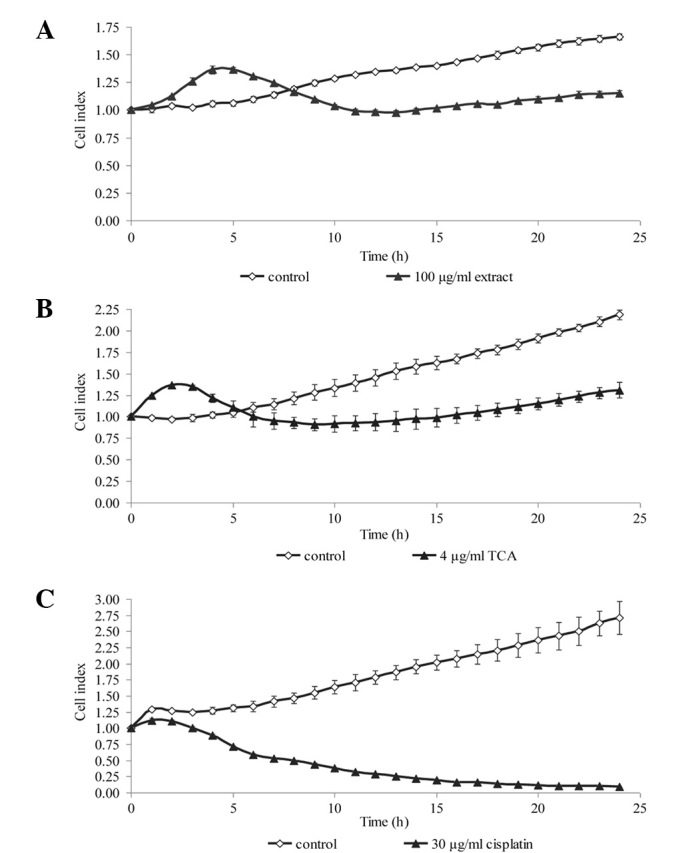
Growth kinetics of HK1 human nasopharyngeal carcinoma (NPC) cells treated with (A) methanol extract of *Cinnamomum burmannii* Blume stem bark, (B) *trans*-cinnamaldehyde (TCA) and (C) cisplatin, determined by the xCELLigence assay.

**Figure 4:**
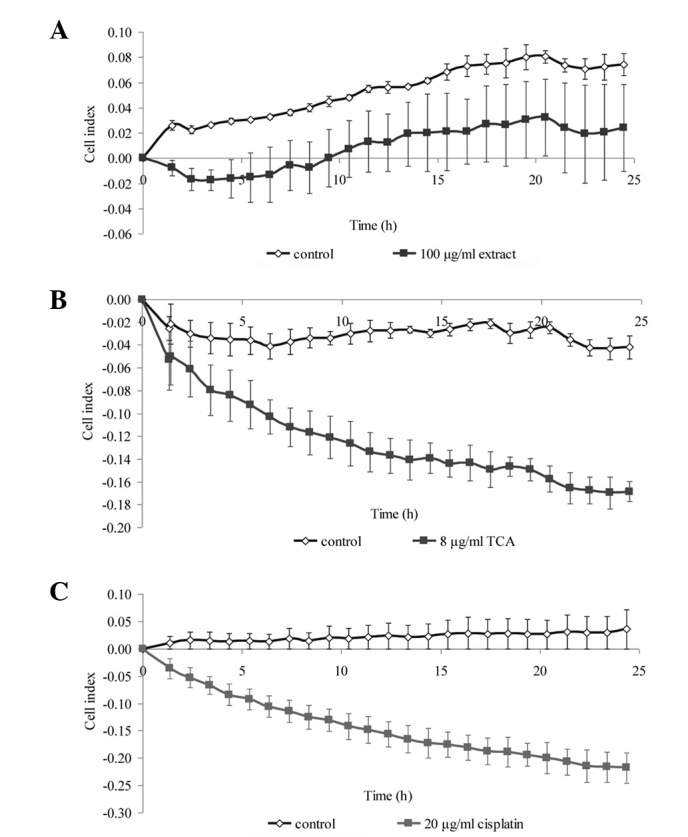
Growth kinetics of C666-1 human nasopharyngeal carcinoma (NPC) cells treated with (A) methanol extract of *Cinnamomum burmannii* Blume stem bark, (B) *trans*-cinnamaldehyde (TCA) and (C) cisplatin, determined by the xCELLigence assay.

To confirm the results from the viability assay, real-time cell proliferation was monitored using the xCELLigence assay, whereby the cell index generated represents growth over time. We selected one concentration from the MTS cell viability assay that produced a significant reduction in cell viability. Within 24 h of treatment, the growth kinetics of the NPC cells was noticeably decreased compared with that of the untreated control (Fig. 3 and 4). The growth kinetics of the cells treated with cisplatin is shown as a positive control.

**Figure 5:**
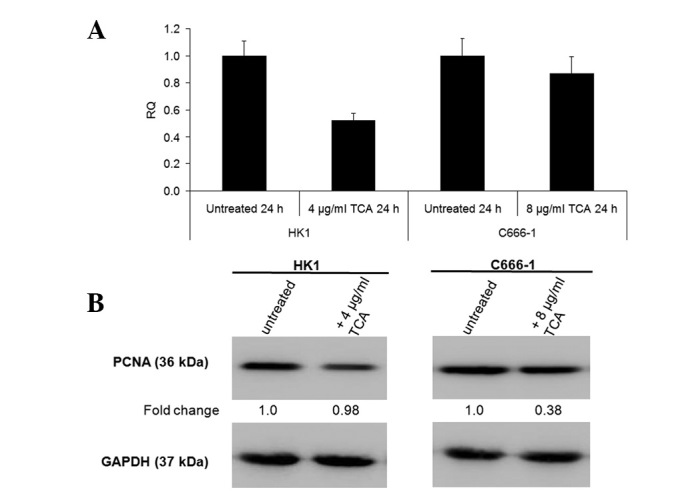
Proliferation studies on the effects of *trans*-cinnamaldehyde on NPC cells performed using (A) qRT-PCR of Ki67 and (B) western blotting with anti-PCNA. *Trans*-cinnamaldehyde caused downregulation of Ki67 and PCNA in NPC cells. qRT-PCR results were expressed as relative quantification (RQ) to the untreated cells following normalization to the endogenous controls, ACTB and PPIA. GAPDH served as an internal control in western blotting. NPC, nasopharyngeal carcinoma; qRT-PCR, quantitative real time polymerase chain reaction; PCNA, proliferating cell nuclear antigen; ACTB, β-actin; PPIA, peptidylprolyl isomerase A; GAPDH, glyceraldehyde 3-phosphate dehydrogenase.

Since TCA, rather than the methanol extract, exhibited greater efficacy at inhibiting growth, all further experiments conducted concerned TCA only. We further verified the ability of TCA to inhibit growth by demonstrating that it decreased the mRNA levels of Ki67 proliferation antigen and protein levels of PCNA (Fig. 5).

### Effects of TCA on morphological changes

**Figure 6:**
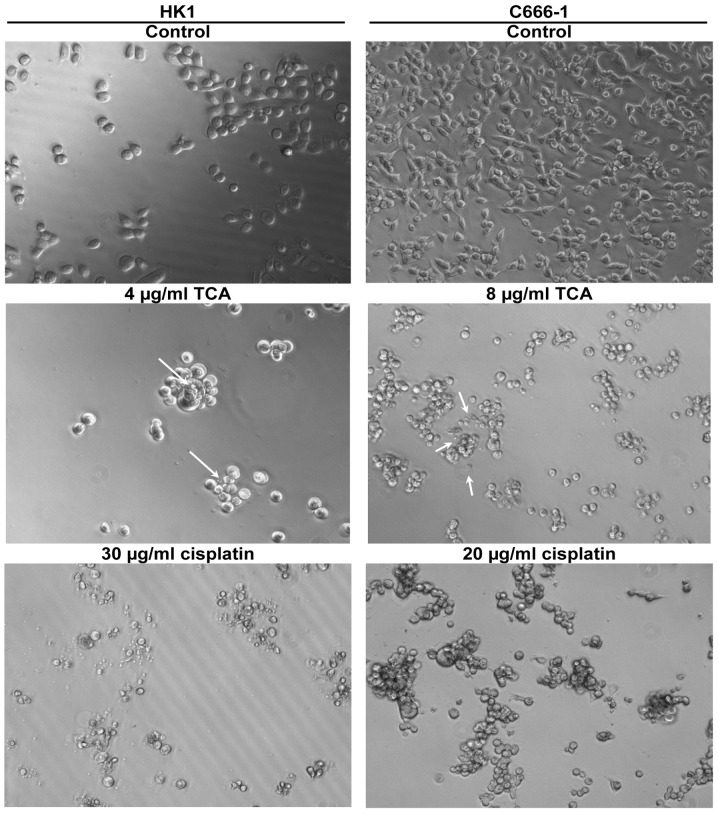
Morphological changes to HK1 and C666-1 nasopharyngeal carcinoma (NPC) cells observed under an inverted microscope after 24 h incubation with *trans*-cinnamaldehyde (TCA) and cisplatin. White arrows indicate distinct morphological characteristics of apoptosis. Representative images are shown (magnification, ×100)

We examined the morphologic characteristics associated with growth inhibition (Fig. 6). HK1 are adherent cells. It was evident that treatment with the methanol extract and TCA within 24 h caused characteristic features of apoptosis, including loss of adherence and roundness in shape. In the C666-1 cell line, control cells were flattened and adherent with few rounded cells. Treated cells detached from the plates to become floating aggregates. These morphological changes were in agreement with the decreased cell index determined in the xCELLigence assay (Fig. 3 and 4). Plasma membrane blebbing and debris, possibly apoptotic bodies, in the culture medium were also apparent. The morphological changes associated with cisplatin treatment were used for comparison. Cisplatin is used in cancer therapy due to its apoptosis-inducing activity.

### TCA induces phosphorylation of histone H2AX and activation of caspase-3/7

**Figure 7:**
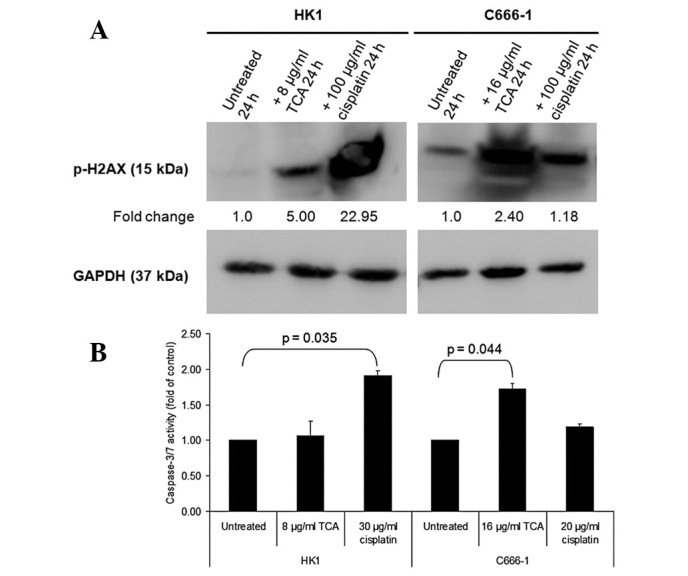
Expression of apoptosis-related proteins in NPC cells. (A) DNA damage as a result of exposure to *trans*-cinnamaldehyde was confirmed by western blotting of phosphorylated H2AX. GAPDH was used as an internal control. (B) Activation of caspase-3/7 in HK1 and C666-1 cells. T*rans*-cinnamaldehyde significantly increased the level of caspase-3/7 activation in C666-1 cells compared with the control group. Data presented are the mean ± SD. P≤0.05 was considered to indicate statistical significance. NPC, nasopharyngeal carcinoma; GAPDH, glyceraldehyde 3-phosphate dehydrogenase; SD, standard deviation.

To elucidate the mechanism of apoptosis, we analyzed the phosphorylation of histone H2AX and changes in caspase-3/7 activity. Clear differences between TCA-treated NPC cells and control cultures were observed with regard to the phosphorylation of histone H2AX, a marker of DNA damage (Fig. 7A). In addition, cisplatin, a drug that crosslinks DNA and interferes with DNA replication, markedly increased H2AX phosphorylation. Fig. 7B shows that treatment with ≥8 *μ*g/ml TCA activated caspase-3/7. A considerable increase in caspase-3/7 activation was noted in C666-1 cells.

### TCA contributes to apoptosis

**Figure 8:**
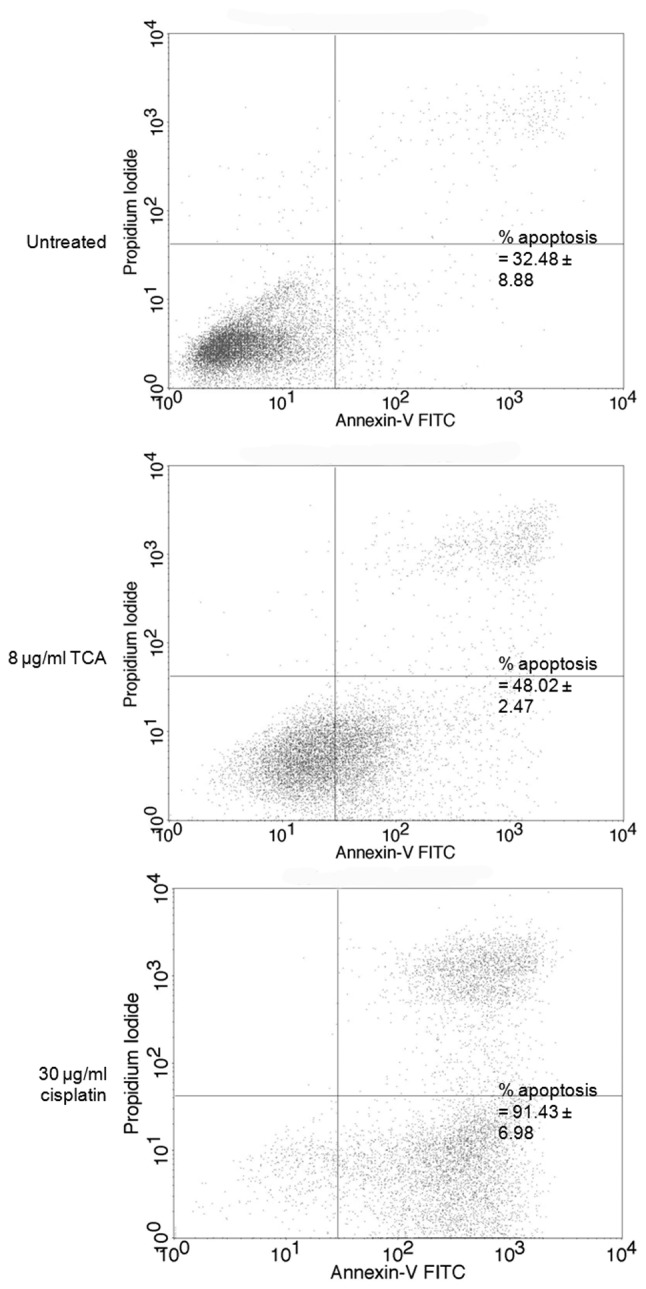
Flow cytometry analysis using annexin V-fluorescein isothiocyanate (FITC)/propidium iodide (PI) double staining to measure apoptosis. HK1 cells were exposed to test compounds 24 h before analysis. The lower right and upper right quadrants represent cells undergoing apoptosis. Images shown are representatives of three independent experiments. The average percentage of total apoptotic cells is shown. TCA, *trans*-cinnamaldehyde.

To confirm the phenomenon of apoptosis, we performed an apoptosis assay by flow cytometry. Approximately 16% of cells underwent apoptosis following correction of background apoptosis, compared with the control (Fig. 8). Cisplatin, used for NPC chemotherapy, was used for clear comparison of apoptosis induction.

### TCA exhibits synergistic effects in combination with cisplatin

**Figure 9:**
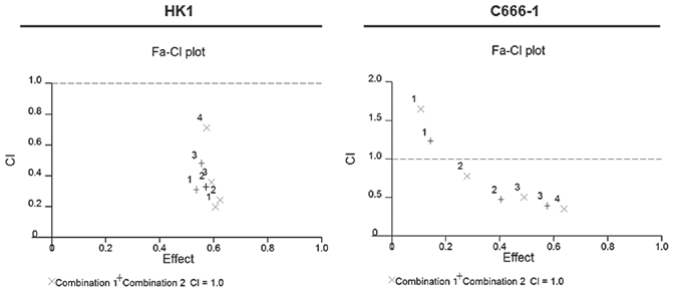
Fa-CI plots for HK1 and C666-1 cells revealed a synergistic cytotoxic effect for simultaneous treatment with *trans*-cinnamaldehyde and cisplatin. In the Fa-CI plot, the dashed line [combination index (CI) =1] indicates an additive reaction between the two substances. Values above and below this dashed line imply antagonism and synergism, respectively.

Using CalcuSyn software, we determined the CI to ascertain synergism (CI <1), antagonism (CI >1) or additive effect (CI =1). The CI values are presented in Table II. The CI method (17) revealed that the simultaneous combination of TCA and cisplatin produces synergistic anti-proliferative effects (Fig. 9).

### Nitric oxide inhibition activity

The nitric oxide-scavenging activity of the methanol extract and TCA were examined using sodium nitroprusside as a nitric oxide donor *in vitro*. The samples demonstrated concentration-dependent inhibitory activity against the nitric oxide radical. The IC_50_ values for curcumin (positive control), methanol extract and TCA were 32.45±1.56, 30.67±1.12 and 24.74±1.25 *μ*g/ml, respectively.

## Discussion

There is a requirement to ensure the quality control of herbal medicinal plant products by using modern techniques and applying suitable standards, since diverse medicinal extracts from the same plant material may vary with respect to their bioactive contents and consequently their therapeutic effects. Therefore, the objective of the GC-MS in this study was to standardize the methanol extract of *C. burmannii* stem bark using TCA as a marker, before proceeding with the anti-proliferative studies. TCA was selected as a marker for this study since it has been identified as one of the bioactive compounds in the *Cinnamomum* species (6). The GC-MS method was suitable for separating and quantifying TCA from the methanol extract.

The present study demonstrated that the methanol extract of *C. burmannii* stem bark and TCA impair NPC cell proliferation. The anti-proliferative activity of TCA in the NPC cells was greater than that of the methanol extract, suggesting that TCA was responsible for the cytotoxicity exhibited by the methanol extract (Table I). There was a moderate degree of selectivity against NPC cell lines compared with human skin keratinocytes, with the anti-proliferative activity of TCA two- to six-fold higher in NPC cells (Table I). Apoptosis is characterized by morphological changes, including cell shrinkage, membrane blebbing, DNA fragmentation, activation of caspases and cell breakdown into apoptotic bodies (19). H2AX, a histone H2A variant becomes phosphorylated at serine 139 to form γH2AX upon DNA double-strand breakage (20). A number of drugs and cytotoxic agents are associated with the formation of γH2AX (21). H2AX phosphorylation is considered to be the earliest known marker of DNA damage (22) and its detection provides a more sensitive and efficient measure of DNA damage than other techniques (20). In the current study, we detected that phosphorylated H2AX (Ser 139) was much more upregulated in the TCA-treated groups than in the untreated control (Fig. 7A). Caspases are proteases involved in the execution of apoptotic cell death (23). The activation of caspase-3/7 demonstrated in the NPC cells in our study (Fig. 7B) suggests a caspase-dependent mechanism for TCA. Collectively with the morphologic observations (Fig. 6) and flow cytometry apoptosis assay (Fig. 8), we infer that TCA induces apoptosis in NPC cells.

The concomitant treatment of cultured NPC cells with cisplatin and the flavonoid, quercetin, has been shown to enhance cytotoxic effects (24). In our study, we successfully demonstrated that TCA, a phenolic acid, is able to enhance the anti-proliferative effect of cisplatin in cultured NPC cells (Fig. 9 and Table II). In nature, TCA and its derivatives serve as important intermediates for the biosynthesis of flavonoids.

The nitric oxide-scavenging activities of the extracts may be attributed to the phenolic contents in the extracts, including TCA. TCA and its derivatives have been reported to inhibit nitric oxide production(25). Phenolic compounds are known to suppress nitric oxide production and also scavenge nitric oxide in an acellular system using sodium nitroprusside under physiological conditions at a micromolar range (26). The Environmental Protection Agency (EPA) has not classified nitrogen oxides for potential carcinogenicity. Nevertheless, it has been reported that exposure to high levels of nitric oxide may result in membrane damage, since nitric oxide reacts with oxygen to form nitrogen dioxide, which in biological systems initiates the auto-oxidation of fatty acids in lipid membranes (27). The nitric oxide-scavenging activity of the extracts indicates that infusions of *C. burmannii* may serve as therapeutic agents for scavenging free radicals and thereby inhibit the pathological conditions caused by excessive generation of free radicals and their oxidation products.

In conclusion, compared with conventional drugs, including cisplatin, the lower toxicity of TCA in normal cells, whilst maintaining or improving the anti-proliferative effect in NPC cells, may contribute to its potential use and benefit as a herbal medicinal product. TCA not only inhibits cell proliferation but also induces characteristic features of apoptosis. Additionally, TCA synergized with cisplatin to produce anti-proliferative effects in NPC cells. *C. burmannii* stem bark extracts and TCA are able to trap and scavenge free radicals, which may be a mechanism involved in their therapeutic effects. These results merit further investigation into the modulation of anti-proliferative action and/or induction of apoptosis.

## Figures and Tables

**Table I:** IC_50_ values for the anti-proliferative activity of cinnamon methanol extract, *trans*-cinnamaldehyde and cisplatin against cancer and normal cells.

	IC_50_ value (*μ*g/ml)		
Cell line	Extract	*Trans*-cinnamaldehyde	Cisplatin
HK1	108.32±3.43	2.94±0.17	26.89±0.26
C666-1	224.32±3.17	6.30±0.74	>60
HaCaT	320.29±30.41	12.92±0.40	7.65±0.16

IC_50_ values were determined in proliferation assays as specified in Materials and methods. Results are the mean ± standard deviation (SD).

**Table II:** Description of CI values for each fraction of cells affected and the corresponding DRI.

NPC cell line	Non-fixed ratio combination	Fa	CI	Description	DRI^a^	
					TCA	Cisplatin
HK1	1	0.606	0.199	Strong synergism	15.824	7.365
		0.624	0.245	Strong synergism	16.630	5.417
		0.592	0.358	Synergism	15.232	3.415
		0.574	0.713	Moderate synergism	14.512	1.552
	2	0.536	0.311	Synergism	8.748	5.086
		0.581	0.334	Synergism	9.858	4.294
		0.553	0.469	Synergism	9.149	2.779
C666-1	1	0.107	1.652	Antagonism	13.318	0.634
		0.279	0.779	Moderate synergism	21.684	1.365
		0.490	0.504	Synergism	31.649	2.118
		0.639	0.355	Synergism	40.803	3.023
	2	0.145	1.218	Moderate antagonism	5.129	0.977
		0.405	0.472	Synergism	9.141	2.758
		0.574	0.392	Synergism	12.143	3.228

a DRI values >1 are beneficial and the greater the DRI values, the greater the dose reduction for a given therapeutic effect (17). CI, combination index; DRI, dose reduction index; NPC, nasopharyngeal carcinoma; TCA, *trans*-cinnamaldehyde.
